# What does it take to consent to islet cell xenotransplantation?: Insights from an interview study with type 1 diabetes patients and review of the literature

**DOI:** 10.1186/s12910-021-00607-5

**Published:** 2021-04-01

**Authors:** Johannes Kögel, Sandra Thiersch, Barbara Ludwig, Jochen Seissler, Georg Marckmann

**Affiliations:** 1 Institute of Ethics, History and Theory of Medicine, Ludwig‐Maximilians‐Universität München, Munich, Germany; 2 University Hospital Carl Gustav Carus, Technische Universität Dresden, Dresden, Germany; 3 Medizinische Klinik Und Poliklinik IV, Diabetes Zentrum, Klinikum der Ludwig‐Maximilians‐Universität München, Munich, Germany

**Keywords:** Xenotransplantation, Porcine islet cells, Diabetes, Informed consent, Qualitative interviews, Patient evaluation

## Abstract

**Background:**

The transplantation of porcine islet cells provides a new potential therapy to treat patients with type 1 diabetes mellitus (T1DM). Compared to other biomedical technologies, xenotransplantation stands out in terms of its involvement of animals as graft sources, as well as the possible transmission of infectious diseases. As these aspects are especially relevant for potential xenotransplantation recipients, it is important to assess their opinion regarding this technology, in particular in terms of the requirements that should be met in the informed consent process for xenotransplantation.

**Methods:**

We conducted qualitative interviews with seven T1DM patients to assess their information needs prior to xenotransplantation. Before the interview, the participants received a model informed consent form for a clinical trial with porcine islet cells transplantation. The interviews were transcribed and analysed using qualitative content analysis.

**Results:**

In the interviews, we identified several requirements that are crucial for patients with T1DM in order to consider xenotransplantation as a potential treatment option: therapy-related requirements, professional care and supervision, successful behaviour and attitude management, improving quality of life, and managing control/self-determination challenges. Regarding the informed consent form, several of the participants’ questions remained open and should be addressed in more detail. The interviewees stressed the importance of personal consultations.

**Conclusions:**

To become a sustainable therapeutic option, patients especially expected an improved diabetes control and a reduction of diabetes-related burdens. Health-related aspects prove to be pivotal for diabetic patients when considering porcine islet cell transplantation. The use of pigs as source for organ retrievals was not considered as problematic.

**Supplementary Information:**

The online version contains supplementary material available at 10.1186/s12910-021-00607-5.

## Background

Xenotransplantation differs from other biomedical therapies especially in two respects. First, as animals are used to provide organs, tissues or cells, the question arises which role animals shall play in our society and if it can be ethically justified to use them as a source for organ-replacement therapies. Second, xenotransplantation bears the small, but not completely excludable risk of the transmission of viruses from animals to the recipient. The risk of infectious diseases makes xenotransplantation a relevant issue also to people beyond the xenograft recipient. This peculiarity of xenotransplantation is reflected in the ethical literature, also regarding its relevance for informed consent to xenotransplantation therapies [1–4]. As any member of society is potentially at risk of acquiring a xenogeneic infection, the informed consent of the patient who shall receive the xenograft is not enough: xenotransplantation becomes a societal matter [1]. Hence, a “collective informed consent” [2] or a “community consent” [5] have been suggested. As the community at risk is the global community, xenotransplantation also becomes a matter of global justice [3, 6].

However, for potential xenotransplantation candidates, more practical aspects are of primary interest [7]. They are concerned with questions regarding how a xenograft will affect their well-being, identity and self-perception, as well as how others will perceive them, including fear of stigmatisation. In addition, the effects on partners and family have to be considered. Besides these psychosocial aspects, patients have a high interest in maintaining a good quality of life and reduce the diabetes-associated burden.

Among the various types of xenotransplantation, the transplantation of islet cells is the only form of cell or tissue transplantation that has seen clinical implementations [8, 9]. As it is likely that Germany will see its first islet xenotransplantation within the near future, we have chosen to interview potential candidates, i.e. patients with T1DM, to assess their attitudes to this potential therapy.

Before presenting and discussing the results of the interview study, we shall give an overview of socio-empirical studies with potential and actual patients of islet cell xenotransplantation, followed by a brief review of the literature about informed consent in xenotransplantation cases.

### Attitudes and opinions of potential and actual xenograft recipients

Several studies have examined the views of diabetes patients who qualify as potential recipients of islet cell xenotransplantation on this potential diabetes therapy. There have been a few surveys and one interview study with potential xenograft recipients and two interview studies with actual xenotransplantation patients. All study participants were T1DM patients.

Among the participants of a survey study with diabetes patients (type 1 and 2), 79% would accept the transplantation of porcine islet cells [10]. Of those, 40% could imagine facing psychological problems due to having porcine cells in their body. Another survey with T1DM patients reported an acceptance rate of 52% [11]. However, a majority (70.5%) opted against xenotransplantation when they learned about the potential transmission of unknown diseases, the risks of immunosuppression, and that the transplantation might not result in complete insulin independence.

A questionnaire study about four alternative therapies for diabetes was conducted among 85 patients with T1DM [12]. In addition to islet cell xenotransplantation, allogeneic islet transplantation, DNA vaccination, and induced pluripotent stem cell therapy (IPS) were presented. The preferred treatment was IPS (77.3%), followed by allogeneic islet transplantation (54.3%), xenotransplantation ranked third (52.2%) before DNA vaccination (41.3%). The participants reported the prospect of being insulin independent as the most important motivator for accepting a new therapy, ahead of avoiding hypoglycaemia.

A recent study among 47 patients with T1DM with a similar research design compared several forms of allogeneic islet transplantation, IPS and (multiple and single) islet xenotransplantation [13]. While 83.3% of the participants would accept IPS, 66.7% would accept single and 46.3% multiple xenotransplantation. The single encapsulated form of allogeneic islet transplantation received the highest acceptance rate of 50%. Again, the majority of patients aimed to become independent of insulin. At the same time, many opted against multiple transplantations.

In a survey with a total of 84 patients from a transplantation outpatient clinic, 65% would accept a xenograft [14]. The acceptance rate among the 27 T1DM patients from this cohort was 85%. 66.7% could imagine the transplantation of porcine islet cells. The majority expressed concerns regarding the transmission of diseases or genetic material, the side effects of immunosuppression, psychological stress, and third-party perception.

The variable results of the surveys may have been caused by differences in the research design and (the wording of) the questions asked, the information given beforehand, etc. Also regional or cultural differences may be at play, as well as sociodemographic factors like age, educational level, or income. Independent of the underlying disease, the severity of the disease, risk assessment, and compromised quality of life are major factors in determining patients’ attitudes [15].

In an interview study with nine T1DM patients with renal failure, the source of the transplant (whether human or animal) hardly played a role [16]. Of primary importance was the functionality of the graft. The priorities of these patients may be related to the progression and severity of their diabetes, which has led to secondary complications. At this stage of the disease, the source of a potential transplant becomes less relevant.

While there have been a few clinical studies with porcine islet cell transplantations [17], only two have examined issues beyond the medical field. In Sweden, eight patients with diabetes and kidney transplantation were injected with porcine foetal islets between 1990 and 1993 [8]. Sarah Lundin examined the experiences of the islet recipients [18–20]. As survival was the priority for the recipients, other considerations became secondary. Hence, they found various ways to justify the use of animal transplants [18, 19]. Patients have “the desire to be healthy, natural, and normal” and for that purpose they are willing to accept an “unnatural technique”[20]. Some transplant patients also felt pressure regarding the “technological imperative”, i.e. having to seek biotechnological treatment in case one exists [19, 20]. Nevertheless, patients reported feeling uneasy about crossing “boundaries of nature” [18]. Given the fluidity of the cells, the recipients did not know where they were, whether they were still in the body, and what they were or may be doing. This uncertainty and loss of control was found as a main psychological challenge for the xenograft recipients [20].

A study in Mexico with ten adolescents with diabetes who received porcine islet cells was published in 2005 and included interviews and questionnaires [9]. The islet recipients reported having no serious concerns regarding infectious diseases or identity issues. Their main motivation was to reduce the required injections, not only for health reasons, but also to avoid unpleasant attitudes from their peers. The increased autonomy allowed more time for other things and let them enjoy foods and beverages, which they had to refrain from before. The islets were perceived as a drug rather than a graft. The recipients reported an improved quality of life, which reversely turned into higher depression scores after the graft lost its functionality. Recipients in which the islet cells did not respond reacted with frustration.

### Informed consent to xenotransplantation

An informed consent to xenotransplantation needs to encompass all aspects that also have to be covered for an allotransplantation. In addition, it must contain the two aspects mentioned above: that the organ or tissue comes from an animal (which may implicate certain psychological challenges) and the risk of transmitting zoonoses.

In a consensus statement, the International Xenotransplantation Association (IXA) outlined structure and content of an informed consent form for a xenotransplantation [4]. Based on statements of the Nuffield Council on Bioethics [21] and the U.S. Department of Health and Human Services Secretary’s Advisory Committee on Xenotransplantation [22], the statement identifies twelve major topics that should be covered in the informed consent process:

1. Identification of the study as medical research and statement of voluntary enrolment
2. Description of the patient’s prospective medical condition
3. Portrayal of possible treatment choices/alternatives
4. Participation information (inclusion and exclusion criteria, randomization, etc.)
5. Study procedures (screening visits, assessments, medications, etc.)
6. Information about potential risks (infections, quarantine, failure rates, matters of quality-of-life, etc.)
7. Post-protocol responsibilities of the patient (monitoring, education of family members and partners, agreement to autopsy, etc.)
8. Potential benefits
9. Costs, compensation
10. Confidentiality
11. Contact information
12. Statement of right of withdrawal from the study

Special features of the consent process for a xenotransplantation are the post-protocol responsibilities (point 7). This is due to the possibility of infectious diseases that may be transmitted from animals to humans. The statement also identifies ten responsibilities of xenograft recipients: “(i) regular post-clinical research check-ups; (ii) informing researchers of future changes of address/contact numbers; (iii) timely reporting of all unexplained illnesses; (iv) following present and updated behavioural guidelines with respect to exchanges of body fluids with intimate contacts; (v) no future donations of blood, sperm, or other body fluids or tissues; (vi) autopsy at time of death; (vii) education of family members and intimate contacts about their need to take precautions associated with infectious disease risks—that includes offered educational assistance from the research team; (viii) disclosure to future healthcare providers that subjects have received a xenotransplantation product; (ix) willingness to accept possible isolation and possible quarantine if necessary for public health; and (x) arrangements for assistance in meeting future responsibilities should the subject lose decision-making capacity” [4]. There are some limitations in the withdrawal from the study according to point 12. Participants can withdraw from the study, but cannot circumvent the post-protocol responsibilities. This conundrum can be resolved by informing the participants in the informed consent that “they are waiving any right to withdraw from their later surveillance responsibilities” [4]. As a complete withdrawal of consent is impossible, informed consent is turned into “obligatory ‘contractual agreements’”[23].

## Methods

### Background, sample and model informed consent

In our study, we interviewed seven T1DM patients (see Table 1) to assess which requirements would be necessary for them to consider the transplantation of porcine islet cells. The hypothetical trial involved the transplantation of macroencapsulated porcine islet cells as described by Ludwig et al. [24]. While microencapsulation constitutes of single islet clusters which are covered by semipermeable (mostly alginate-based) membranes and are implanted into the abdomen [25], macroencapsulation involves the housing of a whole islet preparation within a closed container covered by membrane systems. Upon pre-peritoneal implantation, this macroencapsulation device, the “bioartificial pancreas device”, is connected to subcutaneous fixed ports for external oxygen supply [24].

**Table 1:** Basic characteristics of the interviewees (names haven been pseudonymized)

Interviewee, age	Diabetes history	Therapy (history and assessment)
Mr. A (mid 40s)	Since adolescence; has used insulin from pigs and cows	Very happy with insulin pump; allows for flexibility in his work routine
Mr. B (50)	Since birth; has used insulin from pigs and cows	Using insulin patch pump; excited about continuous glucose monitor **(**CGM), but not paid for by health insurance
Mrs. C (late 50s)	Since 2013	Happy with multiple daily injections (MDI) therapy
Mr. D (30)	Since 2009	Using (tethered) insulin pump (due to problems with patch pump); appreciates flexibility in virtue of the pump
Mr. E (late 20s)	Since 2007	Happy with MDI
Mrs. F (late 40s)	Since age 13; has used insulin from pigs and cows	Very happy with using insulin pump and CGM
Mrs. G (60)	Since age 12; has used insulin from pigs and cows	Happy with insulin pump (before pump: often unconscious); occasionally additional injections necessary; stent (due to diabetes)

The participants were recruited via the diabetes outpatient clinic of the University of Munich hospital. For basic patient characteristics see Table 1. Before the interviews, the study participants confirmed their informed consent in a written form.

All methods included in the interview study were carried out in accordance with the Declaration of Helsinki. The study received prior ethical approval by the Ethics Committee of the Medical Faculty of the Ludwig Maximilian University of Munich (project number 017-12).

To assess potential xenotransplantation recipients’ opinions as realistically as possible, we provided the interviewees in our study with a model informed consent form for a potential clinical trial of porcine islet cell xenotransplantation. It contained all the points, as outlined by the IXA, listed above.

The form features details on the operators, funders and participating parties of the study, details on content, objective, structure, circumstances and proceedings of the transplantation, potential risks and benefits for the patients, and terms and conditions of participation (Additional file 1). The entire original form (in German) can be accessed upon request from the corresponding author.

### Data collecting and analysing methods

The interviews were semi-structured and followed a topic guide (Additional file 2) consisting of questions regarding the patients’ evaluation of xenotransplantation in general, assessment of background, process and implications of the study, terms of participation, and feedback regarding the model informed consent.

All interviews were conducted at the Institute of Ethics, Theory and History of Medicine in Munich between November 2014 and January 2015. The duration of the interviews was between 33 and 127 min. The considerable variation may be partly caused by different levels of scrutiny invested in reading the provided information by the participants.

The interviews were transcribed verbatim. The interviews were held in German. All quotes from the interviews were translated into English by the authors.

The anonymized transcripts were analysed using qualitative content analysis [26] which can comprise of various techniques of coding, categorizing, or typifying. The interviewees’ accounts rendered substantial insights on the patients’ hold of their experiences with diabetes and their assessments of their living conditions, respectively their quality of life. To utilize these descriptions for a substantive evaluation of xenotransplantation we decided to make use of these passages without applying a coding frame from the beginning. To catalyse these accounts in a best possible way without restricting them to the points stressed in the model consent form we used open coding as developed by Grounded Theory methodology [27, 28]. In a second step codes were clustered in categories, leading to five key categories that are outlined in the following section.

## Results

The aim of the study was to investigate under which conditions patients with T1DM would participate in a specific trial of islet xenotransplantation and what they expect from the informed consent process. One week prior to the interviews, we provided the study participants with a model informed consent form. The form included information regarding xenotransplantation in general and its implications and possible risks and benefits of the proposed trial. The model informed consent form including the description of the trial was based on an actual study protocol that has been under evaluation for clinical application.

The T1DM patients’ statements on what they considered relevant for potential trial participation can be differentiated into five key categories: therapy-related requirements, professional care and supervision, successful behaviour and attitude management, granting quality of life, and managing control/self-determination challenges.

Finally, the participants’ recommendations regarding the informed consent form are outlined briefly.

### Therapy-related requirements

The study participants saw porcine xenografts as a last option in the case of failed standard therapies. As long as conventional therapeutic strategies work for the interviewees, they reported to have no reason to change it. As some had made the experience already of having changed from MDI to an insulin pump, they regarded xenotransplantation as a potential alternative at some point in the future.

In case of receiving a xenograft, the interviewees considered it important not to have to rely on immunosuppressives (this point is stressed in the patient information, but has been spelled out by an interviewee, nevertheless) and to be independent from additional insulin.

The kind and frequency of invasive procedures that would be necessary for refilling the boxes with the islet cells was also reported to be important. Two interviewees found it acceptable to have a refill procedure done once a year, one interviewee found every two years acceptable, and another interviewee found it acceptable to get a refill two times a year. If the refills were possible without surgery, e.g. with a syringe, refill procedures would be more accepted.

### Professional care and supervision

As indicated in the patient information, there would be various specialists that are involved in the study: first and foremost, the medical staff, and a psychologist for consultations. The interviewees found it important to have diabetes and transplant specialists on board; some also wanted to involve their general practitioner as someone they can trust. Otherwise, trust needs to be established between the patient and the physicians in charge, as one interviewee stated:

“I think it is very very important that there is some relationship of trust, because it really has an impact on your life” (Mr. E).

Some participants opted to include a psychological consultant; others mentioned that they would not mind her presence. One interviewee would have appreciated psychological support in order to assess whether she was mentally prepared for transplantation.

The interviewees also found it important that the medical and psychological staff would be present and approachable not only for pre-study consultations, but also during the study and afterwards, during the post-trial monitoring. It was seen as important that

“the patients know that they are in good hands; that there is a really personal care” (Mr. E).

Furthermore, the potential xenotransplantation patients expected transparency from the physicians and consultants in charge. They wanted to be informed regarding the current state of research, the functioning of the porcine islet cells and physiological implications, as well as possible worst case scenarios. They found it especially important that the consultants are honest regarding potential risks and benefits involved, as well as about possible alternative treatments and their respective prospects.

Having met various physicians over the course of her life, one interviewee emphasized her appreciation for those who were able to alleviate someone’s fears and treat someone as a human.

In general, most interviewees trusted the scientific and clinical staff, also when it comes to making sure that there won’t be any risks regarding infections or other diseases.

### Successful behaviour and attitude management

The interviewees figured that transplantation would require changes or adjustments of beliefs or opinions they had held or in the routines they had followed in the past. At the same time some issues were not regarded as problematic.

None of the interviewees considered the use of animals as ethically problematic. Some had already used insulin from pigs or cows in the past. As pigs are used for meat production, their use as organ source posed no problem to the interviewees, while the use of other animals, e.g. protected species, was considered unacceptable. Acknowledging meat consumption in our society puts xenotransplantation into perspective, as one interviewee recognized:

“Eventually, I think, that’s a societal question: we eat a lot of meat and do not think about it” (Mr. E).

Recalling the time they used pig insulin, one interviewee reported:

“I was feeling way better due to the insulin, therefore I didn’t think about it” (Mrs. F).

While the use of animals as such is not seen as a problem, the proportionality between the number of organ sources and recipients was questioned, as one islet filling would require about three to four pigs.

“Just because of one life, which can be perpetuated in some other way, three pigs need to die. That lies heavy on my shoulders” (Mr. A).

Under these conditions, the interviewee would expect the islet cells to last for at least ten years.

The interviewees also did not regard islet cells as playing a relevant role for the recipient’s identity. Rather, they found it important to have a stable living environment that can accustom required changes in the patient’s life and that would allow them to cope with possible hardships. Deemed important in that regard was “being settled”, meaning having a stable place to live and having completed one’s family planning.

One interviewee would have to change her advance directive to allow for an autopsy, which she would be willing to do in case of study participation.

For the interviewees, one of the most relevant points would be whether the xenotransplantation and its requirements can be integrated into one’s daily routine and everyday life. One interviewee had concerns regarding her workplace, in that she would have to be absent from work for a few days every year for the refill procedures. Others saw the frequent act of connecting the device to the oxygen supply as quite obstructive, while others saw no difference to what they had been doing thus far:

“I need to do measurements every day; I need to do injections every day, anyway. That’s a ritual you get used to” (Mrs. C).

Interviewees sought more detailed information regarding how the xenotransplantation would affect their everyday life:

“How long do I have to refrain from sports? How long do I have to stay away from the sauna? Do I need to be cautious about my diet or about some sports activities” (Mr. D)?

### Granting quality of life

There are several aspects affecting one’s quality of life that played a role in the interviewees’ accounts. For once, their mobility needs to be maintained. Quarterly check-ups were still regarded as manageable by most interviewees. Having diabetes, the interviewees reported they were used to frequent or regular visits at their physicians. According to one interviewee, participating in xenotransplantation would require living near the study centre, as the frequent visits would make long-distance travel quite strenuous. As long as changing one’s place of living is not a matter of concern, it did not appear to be a problem for the interviewees. Nevertheless, one would need to think about it before opting for a xenotransplantation.

Also relevant to one’s mobility is the oxygen device, which needs to be plugged in every 24 h. Some considered this to be unproblematic, while others saw it as a major infringement on their mobility. The device should be as small as possible to keep the mobility restrictions to a minimum.

One interviewee who liked bicycling and mountaineering ruled out the possibility of an artificial pancreas device, because according to the informed consent (IC) form there would be too many requirements preventing him from doing these activities and therefore, he would not choose a xenotransplantation. Especially for those who enjoy traveling, hiking, bicycling, or camping, the dependency on electricity (charging the oxygen device) or on devices that use batteries, which need to be changed or recharged, could compromise these activities.

Additional time restrictions that would be caused were mentioned: Having to do measurements every four hours and going to the hospital for frequent check-ups during the monitoring phase would add to the fair amount of time that xenotransplantation and pre-study consultations already require.

Also, comfort needs to be granted. One interviewee ruled out carrying an islet box as he was used to sleeping on his stomach and saw a box as compromising his sleeping habits and hence his quality of life.

A further requirement is to make sure that the xenotransplantation will not affect quality of life in terms of career or family. One interviewee saw the possibility of quarantine as something that could affect his job security, because “you cannot go to your employer saying ‘Listen, I am gonna be in quarantine for three weeks!’” (Mr. D).

He also did not want his employer to know about his diabetes in the first place, “because you are being pigeonholed […] until your employer recognizes […] that you are working just as good as a perfectly healthy person does” (Mr. D).

At the same time, sufficient flexibility needs to be assured, as work does not always allow for fixed meal times, one interviewee stressed. In terms of flexibility, the interviewees appreciated the advantage of the insulin pump as opposed to pen injections, as they also allow for a reduced number of meals a day and hence would allow for more balanced and healthier nutrition.

Partners and family may also be affected in case of the transmission of xenogeneic infections. Potentially necessary measures, such as being put under quarantine, need to be reasonable. That was an exclusion criterion for one respondent, as one of her children has a compulsion neurosis. Another interviewee said quarantine would not be a possibility for his partner, as she would lose her job. Also other interviewees formulated concerns regarding their partners’ jobs. Besides that, no interviewee saw any problems regarding their partner’s or family’s dissent in case they should want xenotransplantation.

A precondition, of course, is that family planning has been completed, as participation in the study requires refraining from reproduction for at least five years after the transplantation. For most interviewees, family planning was off the table already. For one younger interviewee, it was said to be okay to refrain from reproduction for up to five years, but he would not be willing to commit to give it up completely. Another interviewee had plans to start a family and, consequently, ruled out xenotransplantation.

Apart from the workplace, the acceptance of xenotransplantation by society was not regarded as a problem for the interviewees, as they have not experienced discrimination in any way due to their diabetes. Where or how a diabetic gets his daily insulin is not necessarily known by others anyway, one interviewee explained.

Interviewees also requested to have some kind of security in case the xenogeneic therapy fails, financially and medically. There would need to be something you can fall back on in terms of therapy and some kind of insurance that covers expenses that may arise from the transplantation or its failure.

### Managing control/self-determination challenges

A final cluster of issues raised was control and freedom of decision. What interviewees disliked about the islet box was that any control or possibility of regulation would be out of their hands, unlike in the case of the insulin pump, for example. If the device, the bioartificial pancreas, malfunctions and releases too much insulin at once, you would not be able to regulate. At the same time, the box is supposed to be optically unobtrusive.

The control aspect is also prevalent given the unknown risks and potential infections. One interviewee was very sceptical about xenotransplantation. As she had been living with her diabetes for most of her life and has managed to deal with it, the prospect of any risks accompanying the transplantation, in particular regarding unknown viruses or anything of that kind, appeared appalling to her.

“I’ll have that transplantation and may catch something that is unfamiliar. I would be a real guinea pig. For ME personally, I don’t have a good feeling about it” (Mr. F).

One interviewee related this uncertainty to the ethical issue of putting others at risk, given the possibility of infectious diseases:

“this could be a reason to say ‘No, I can’t do it’ out of responsibility for humankind” (Mrs. G).

To maintain as much freedom of choice as possible interviewees deemed it to be necessary to get all the knowledge necessary or available when it comes to the medical procedure and its potential risks and benefits, as well as possible alternatives.

However, given the complexity of xenotransplantation interviewees were aware that they cannot make informed decisions by themselves:

“I am the one who gets surgery and who does this transplantation, but just the risk of infections of some viruses where it is not clear if they exist or not, are—I find—reasons enough to say: This is something where you cannot make a decision on your own” (Mr. E).

One interviewee stressed that the privacy of the decision-making should be preserved. The interviewees therefore did not appreciate public discussions on matters like xenotransplantation, as it could impede one’s decision-making.

Lastly, they would have appreciated to be involved when it comes to the allocation of transplants, more specifically regarding who is supposed to get human transplants and who is supposed to get animal transplants. However, some interviewees regarded this simply as something that is removed from their discretion. Some interviewees also did not categorically prefer human over animal islets. They would opt for what is available and the least complicated procedure.

Only one person would seriously consider xenotransplantation, as the insulin pump had started to fail. Therefore, she was looking for alternatives as a matter of survival. For the other interviewees, their degree of independence was a priority in that their health was already covered by their respective therapies. One interviewee, reflecting on his history of having used various devices for measuring blood sugar and injecting insulin, stated:

“I went through all these things; hence I’m thoroughly a diabetic person. Whereas […] I would say, I’m healthy and have diabetes, by the way” (Mr. B).

Paraphrasing this interviewee, he was considering himself a “healthy diabetic”. However, he had experienced quite the reverse in the past when he had to take meticulous blood measurements for his health insurance provider:

“THAT made me a diabetic again” (Mr. B).

Regarding the potential infringements because of the xenotransplantation (4 h measurements, 24 h oxygen supply, no CGM, jeopardising one's work life), he came to the conclusion: “I’ll be treated to death. With that I’ll DEFINITELY be a living dead diabetic” (Mr. B).

The prospect of still having to inject insulin even with an bioartificial pancreas device would be an exclusion criterion for some, as there would be no gain in freedom or independence. A central requirement for accepting an islet xenotransplantation would be insulin independence.

### Assessment of the informed consent form

As mentioned above, interviewees sought comprehensive information to grant self-determined decision-making:

“The patient has to decide, whether he wants this or that. He should have a FREE choice and COMPLETE information” (Mr. B).

Hence, some more detailed information should be included in the informed consent form. For example:

- It should be stated that family planning should be completed or off the table.
- Alternative therapies, such as stem cell transplantation, should be discussed.
- Detailed information should be provided on the functioning of the Langerhans islets.
- Data of previous or similar studies should be given.

Information that was not present in the informed consent form includes the following points:

- The life expectancy of the cells and possible consequences: How long do islet cells survive in the box? What happens when cells die in the box? Do they have a negative impact on your body?
- Maintaining the devices: How do I keep the devices sterile (when changing the inside of the box, the oxygen supply cables and plugs)? What effort does that take? What happens when there is no oxygen supply for more than 24 h?
- Behaviour in case of emergencies: What about when I have high blood pressure (in that you need to refrain from blood-thinning medication)? What happens when a disease is discovered, but cannot be cured or turns out to be risky? What happens when I have low blood sugar?
- Alternatives: Why not use a continuous glucose monitor (CGM) instead of doing measurements every four hours?
- Monitoring: Why is it necessary to have check-ups even after you stopped using the islet cells (i.e. beyond the five years threshold)?
- Coverage and insurance: What expenses and costs will become necessary after the study? For how long? Compensation of partners or family or others in case of quarantine?
- What are the everyday life restrictions, e.g. how long until I can do sports again, sauna, etc., or restrictions in nutrition?

One interviewee stated that a detailed IC would provide sufficient information for her. Other interviewees stressed the importance of oral consultations as they prefer face-to-face meetings over written forms. The consultation was also deemed important to get a better understanding and because the form is quite objective and impersonal: one interviewee is “not sure, if this form can relieve one’s FEARS” (Mr. E). Therefore, the personal consultation was seen to be vital in this regard in particular.

Because of the limited capacity to process or register spoken words, multiple consultations and repeating the challenges of the transplantation were regarded as being necessary in order to make sure that the decision of the patient is really well informed.

Interviewees also suggested some change of words and formulations for reasons of clarity and comprehension.

## Discussion

Serious health-related reasons are the prerequisite for persons with diabetes to consider the transplantation of porcine islet cells. Otherwise, priority is given to maintain their quality of life with the current therapy. Potential xenotransplantation patients do consider ethical and social aspects like animal use and communicable diseases, but personal health and well-being becomes more important in light of the impact of their illness. This prioritization reflects the findings from Idvall [16] and Lundin [18, 19].

Every diabetes therapy should retain the “healthy diabetic” as a central notion of self-understanding. Maintaining this identity is deemed important by potential xenotransplantation patients. Another interviewee stated that diabetes “simply belongs to me” (Mrs. F). The opposite would be the “diabetic patient”, where the diabetes leads to a noticeable infringement of one’s freedom and independence. To be dependent on something, in this case a particular therapy and technology, presupposes not having to worry too much about things.

Having diabetes, the interviewees are familiar and used to therapeutic consultations and procedures and have gathered a lot of experience in interacting with medical staff and dealing with medical and health-related issues. Due to this familiarity, most interviewees display a great level of trust in medicine and physicians.

Trust and transparency are essential to bridge the gap between autonomy and health. Being aware of the fact that you cannot fully assess the risks of this procedure and need to rely on others, you have to make concessions in terms of control and self-determined decision-making.

One interviewee also acknowledged that the decision for or against xenotransplantation cannot be up to one alone. In this case he had his partner in mind, but it also applies to other people. This turns the matter into a societal decision. Xenotransplantation is seen as a societal question also when it comes to the use of animals for our sake. This question is answered in favour of xenotransplantation, as we already accept the killing of animals for food production.

Given that in the hypothetical xenotransplantation trial that was portrayed in this study the islet cells would be contained in a box, the participants expressed no uncertainty regarding the placement of the cells like in the studies by Lundin. However, the potential transplant recipients did not know if cells are dying and what the consequences would be. As they do not have access to the islet box, they also missed the control over how the cells work. Like in other studies [11–13], the interviewees also expected insulin independence from an artificial pancreas device and not having to inject additional insulin themselves. Hence, there are further issues concerning the loss of control.

The interviewees of this study assumed that the source of the cells, i.e. pigs, would not bother them. Instead, psychological problems may arise from difficulties with the treatment, challenges being imposed upon family and partners, or even the stigmatization as a person with diabetes. The risk of the transmission of a xenogeneic infection caused worries among some interviewees, which is similar to other studies [11, 14]. However, our interviewees were mainly concerned with possible quarantine for family and partners. As the psychological problems raised by Abalovich [10] are not elaborated, it is hard to draw comparisons. In the case that identity crises may occur due to the knowledge that animals are the organ sources, as in the study by Stadlbauer [14], our interviewees did not envisage them. Also in contrast to that, stigmatization by friends and family is not feared, but may become relevant for some at the workplace.

As indicated at the beginning, potential patient interviewees are primarily concerned with their own health. While they take into account the consequences for their partners and family, worries regarding public health are not seen as their responsibility. This task lies upon society or the government [23].

Instead, what clearly comes to the fore in these interviews and what is hardly reflected in the literature so far, are the concerns regarding the impact of xenotransplantation on the interviewees’ routine and everyday life. These include whether they will be able to function in the same way, whether they will be able to pursue sports and outdoor activities that they are used to and which they cherish, whether they will be able to perform their jobs in a normal manner, or if they will need to change their eating habits.

### Limitations

The study contains several limitations. First of all, the study involves only a small number of participants. As the reading of the model informed consent form prior to the interviews was a precondition for participation, recruitment turned out to be more difficult than expected. At the end, only seven participants were willing to complete that task and participated in the study. While the small sample size is not in general at odds with the methodological standards of qualitative research, it may somewhat reduce the generalizability of our findings.

The sample of the study consists of persons that vary in many respects, in particular regarding their age and the progression of their diabetes. More detailed research with respective study groups is encouraged. As xenotransplantation research often involves genetically modified pigs, patients’ opinions regarding this important aspect should also be assessed. While this study is focused on macroencapsulated cell transplantation, alternative technologies, i.e. transplantation of free or microencapsulated islet cells [17], also ought to be discussed. These rather specific studies can be complemented with further studies on xenotransplantation in general as well as with studies assessing public opinion on xenotransplantation [29].

Given the hypothetical character of the scenario discussed, the assessment of the study participant’s account serves best to adjust future informed consent forms and procedures accordingly and must not be overestimated in terms of actual consequences of xenotransplantation for the patients.

## Conclusions

In conclusion, it can be stated that for porcine islet cell transplantation to become a sustainable therapy for diabetes, it has to guarantee the “healthy diabetic”. The patient should be able to continue living her life without having to sustain too many infringements and restrictions. Where measures necessary for the procedure fit within the patients’ regular diabetes regime of check-ups, measurements, injections, etc., a xenotransplantation is conceivable. When additional requirements are due and limitations on things of daily life that are appreciated by the patients are expected, xenotransplantation becomes problematic.

Given the current requirements, most persons with diabetes would consider xenotransplantation only as *ultima ratio*. A comprehensive and comprehensible informed consent form needs to meet all requirements in order to contribute to the potential participants’ decision-making and to make it as well informed and autonomous as possible.

## Supplementary Information

Additional file 1: English summary of model informed consent

Additional file 2: Topic guide for interviews with T1DM patients on the potential transplantation of porcine islet cells

## Data Availability

The data sets generated and analysed during the current study are not publicly available, because a significant proportion of them identify people and organizations. Anonymous subsets are available from the corresponding author on reasonable request.

## Abbreviations

CGM: Continuous glucose monitor
IC: Informed consent
IPS: Induced pluripotent stem cells
MDI: Multiple daily injections
T1DM: Type 1 diabetes mellitus
