# Supplementary material for: What does it take to consent to islet cell xenotransplantation?: Insights from an interview study with type 1 diabetes patients and review of the literature
Source: BMC Med Ethics. 2021 Apr 1;22:37. doi: 10.1186/s12910-021-00607-5 (PMC8015214; doi:10.1186/s12910-021-00607-5)
Supplement: Supplementary file 1 — Additional file 1: English summary of model informed consent [file 12910_2021_607_MOESM1_ESM.docx]

**Additional file 1: Model informed consent (English summary)**

**Who**

Researchers/medical team

- Medical teams of three universities in Germany (cities A, B and C) + participating institutions
- Contact information
- Funding company: XYZ Pharma Incorporated

**What**

Implanting a bioartificial pancreas device with porcine islet cells

- Background of the study
  - Preceding tests with animals
  - Successful test study with human islet cells
  - First test study with porcine islet cells in humans (test group of 20 persons)
- Details on porcine islet cells
  - Source: pigs bred in particular for purposes of transplantation, held under high hygiene standards
  - Porcine endogenous retroviruses (PERV) are existent in all pig species (to a lesser but still existing extent also in the pigs bred for transplantation); infections of humans with PERV cannot be excluded
  - Islet cells are retrieved from the pancreas of dead pigs in a sterile environment and processed in a laboratory to ensure cells void of any contagions
  - For each transplantation islet cells from 2 to 3 pigs are needed
  - islet cells encapsulated in alginate and contained in a cell box
- Details on device, its set-up, mechanism and components
  - Components: cell box, oxygen system, oxygen device
  - Mechanism of the cell box: cell container with semipermeable membrane (ensures that the immune system does not attack the embedded islet cells)
  - Placement of cell box in abdomen, subcutaneously; connecting to exogenous oxygen device every 24 h
- Study phases explained
  - Phase 1: information, check-ups, consultations (medical and psychological), consent
  - Phase 2: operation (transplantation of cell box into abdomen) under general anaesthetic
  - Phase 3: post-operation tests, weekly check-ups at the clinic (for 12 weeks)
  - Phase 4: monitoring (regular check-ups (monthly to annually), including life partner)
- Assessment of the study
  - Approval by Paul Ehrlich Institute
  - Ethics clearance by ethics committee of the Ludwig-Maximilian University Munich

**To whom**

Patients with type 1 diabetes mellitus (T1DM) (n=20)

- Criteria of eligibility
  - Age, able to consent
  - Particular T1DM conditions (treatment history, low HbA1c levels, number of hyperglycaemia, etc.)
  - Agreement to terms of participation listed below

**To what end**

Improved health situation in T1DM patients

Short term: improved control of one’s blood sugar level; less cases of unrecognized hypoglycaemia; less demand of insulin

Long-term: full insulin independence

- Prospects of the treatment
  - The treatment is no “cure” for diabetes
  - Realistic objectives of the treatment are stabilized blood sugar results in combination with a lower total in demand of insulin
  - Due to the containment in the box no immunosuppressive medicaments are necessary
  - The durability of porcine islet cells in human bodies cannot be anticipated at this point in time

**Under which circumstances**

- Participation in the test study presupposes an informative consultation of the potential study participant and his/her partner (Monitoring regime accompanying the study; the necessity of informed consent of the partner; Informing persons that are met regularly, relatives and acquaintances, on the conditions and consequences of xenotransplantation)
- Pre-study check-ups by medical and psychological professionals will be conducted.
- Terms of participation
  - Voluntary participation
  - Guarantee of no repercussions in case of no participation (continuation of regular therapy, retaining transplantation list placing)
  - Right to refrain from study at any time, also after consent to participation has given
  - Consent to (in case of study participation)
- Regular, lifelong monitoring
- Refraining from biological reproduction
- Rendition of (changing) contact information
- Timely reports in case of unforeseen complications
- Disclosure of your condition to present and future sexual partners and family members
- Use of contraception
- Refraining from donations from blood, ovocytes, sperm, breast milk, organs or any other body parts or fluids
- Informing all medical practitioner one gets treatment from and health care insurance
- Health care proxy in case of inability to consent
- Agreement on post-mortem examination
- Caveat: no preceding tests of porcine bioartificial pancreas device in humans! Risks and benefits are hence unknown.
- Alternative therapies
  - Continuation of current therapy (intensive insulin therapy) , which qualifies as an established standard therapy with minimal strains and risks
  - Transplantation of human islet cells
  - Transplantation of human pancreas
- Costs and compensation
  - All expenses are covered by funding agency
  - Insurance via German Drug Law (AMG)
  - Harm or injuries inflicted through the study are liable to compensation which will be assessed through third party reviewers

Data protection and informed consent

- Anonymization (pseudonymization) of necessary personal data
- Safe storage of data, confidentiality granted
- Declaring understanding of provided information and consent to terms and conditions as outlined

**With which consequences**

Assessing benefits and risks

- Potential benefits of study participation (possibility of no occurring benefits at all!)
  - Improved control of blood sugar level, less unrecognized, life-threatening hyperglycaemia
  - Diminished insulin consumption
- Potential risk of study participation
  - Infections (xenozoonoses such as PERV or others); consequences can be quarantine for patients, life partner and/or family members
  - Disturbances of blood sugar control
  - No efficiency: non-functioning cells
  - Restrictions of quality of life
  - Further unforeseen risks cannot be ruled out completely
  - Possible side-effects and risks of surgeries in general, of antibiotics which are administered after the operation, and in combination with the port system
