# Supplementary material for: What does it take to consent to islet cell xenotransplantation?: Insights from an interview study with type 1 diabetes patients and review of the literature
Source: BMC Med Ethics. 2021 Apr 1;22:37. doi: 10.1186/s12910-021-00607-5 (PMC8015214; doi:10.1186/s12910-021-00607-5)
Supplement: Supplementary file 2 — Additional file 2: Topic guide for interviews with T1DM patients on the potential transplantation of porcine islet cells [file 12910_2021_607_MOESM2_ESM.docx]

**Additional file 2: Topic guide for interviews with T1DM patients on the potential transplantation of porcine islet cells**

Please imagine the following scenario: You are being offered to receive a xenograft against your diabetes. In order to participate in this xenotransplantation study, you need to read and consent to the terms outlined in the informed consent form attached.

1. Which points do you consider as positive? Which ones as negative?

2. What is your hold on the outlined advantages?

3. What about the risks? Which ones would you be willing to take and which not?

4. What do you think about the regular check-up visits? What about the daily oxygen supply procedure?

5. How do you evaluate the durability of the bioartificial pancreas device?

6. Does it make a difference to you whether the xenograft is supposed to work as bridging technology or as a permanent solution? Which one?

7. What do you think about pigs being the source for the retrieved islet cells? Are there other animals you could imagine getting insulin from?

8. What do you think about the terms and conditions of participation for the study?

9. How do you feel about life-long monitoring?

10. How do you feel about the monitoring, given that your partner and family need to participate in it, too?

11. Your partner also would need to declare their informed consent. What do you think about your partner’s responsibilities?

12. What if your partner would not be willing to consent?

13. Is it important to you that the transplantation will be accepted by your friends and family?

14. Is it important to you that the transplantation will be accepted by society?

15. What is your opinion on the expenses being covered? On compensation payments?

16. Do you see any responsibility on the side of the patient?

17. Are there persons you want to talk about your decision on participating in the study? If so, who?

18. Would you be willing to opt for a xenotransplantation under the circumstances outlined in the informed consent form? Which changes that would be necessary for you to accept xenotransplantation?

19. Can you imagine a situation in which you rather would be accepting a xenotransplantation?

20. Do you regard xenotransplantation as a viable alternative to allotransplantation? Why/why not?

21. Is there anything else you would like to add?
